# Clinical outcomes in acute pancreatitis with relative bradycardia at fever onset

**DOI:** 10.1097/MD.0000000000027901

**Published:** 2021-11-19

**Authors:** Takeshi Okamoto, Makoto Arashiyama, Kenji Nakamura, Ryosuke Tsugitomi, Katsuyuki Fukuda

**Affiliations:** a Department of Hepato-Biliary-Pancreatic Medicine, Cancer Institute Hospital of Japanese Foundation for Cancer Research, Koto-ku, Tokyo, Japan; b Department of Gastroenterology, St. Luke's International Hospital, Chuo-ku, Tokyo, Japan; c Department of Gastroenterology and Hepatology, Tokyo Medical University, Shinjuku, Tokyo, Japan; d Department of Gastroenterology, Tokyo Dental College, Ichikawa General Hospital, Ichikawa, Chiba, Japan; e Department of Thoracic Medical Oncology, Cancer Institute Hospital of Japanese Foundation for Cancer Research, Koto-ku, Tokyo, Japan.

**Keywords:** heart rate, necrosis, pancreatitis

## Abstract

While some acute pancreatitis (AP) patents with fever do not exhibit a corresponding increase in heart rate, the clinical significance of this phenomenon has not been studied. We investigated the clinical relevance of relative bradycardia (RB) in febrile AP.

A retrospective electronic chart review was conducted on consecutive patients admitted for AP at a tertiary referral center in Japan from January 1, 2010, to May 31, 2018. Presence of RB was determined at the first instance of fever, based on formulas used in previous studies.

Fever at or during admission was observed in 115 patients, of which 33% had RB. Fever was observed at presentation in 48% and within 48 hours in 94% of cases. Etiologies were alcoholic in 48% and gallstones in 17% of cases. RB patients were older (median age: 62 vs 51 years, *P* = .028) but had shorter median postfever lengths of stay (8 vs 12 days, *P* = .003), lower median Ranson scores (1 vs 2, *P* < .001), and were less likely to develop delirium (11% vs 38%, *P* = .002). Nineteen of 21 severe AP cases based on the Ranson score were nonbradycardia (*P* = .011). RB was also associated with lower white blood cell count, C-reactive protein, and lactate levels. On computed tomography, necrosis (*P* = .028) and moderate or severe pancreatitis (*P* = .041) were less frequently observed in patients with RB. There was a significant inverse correlation between RB and the Ranson score (−.305, *P* = .001). While RB was an independent predictors of postfever length of stay (LOS) in multiple regression analysis when the Ranson score was excluded (*P* = .010), it ceased to be significant when the Ranson score was included (*P* = .141).

AP patients with RB at fever onset had milder disease and shorter LOS compared to those with higher heart rates at fever onset. RB may be useful as a simple, early predictor of shorter LOS before the Ranson score can be calculated.

## 1 Introduction

Acute pancreatitis (AP) is an inflammatory disorder of the pancreas. It is mild and self-limited in 80% of patients but severe AP has a reported mortality of 15% to 20%.^[1,2]^ While scoring systems can be useful to predict severity, calculations can be time-consuming and the availability of numerous scoring systems makes comparisons difficult.^[3]^ Some scores such as the Ranson score also include items which must be evaluated 48 hours after admission, delaying prediction of severity.^[4–6]^

Fever is commonly seen in AP but has received surprisingly little attention in the literature. It has been our clinical experience that AP patients with relative bradycardia (RB) tend to have milder disease and shorter length of stay (LOS). We therefore evaluated the relationship between RB at fever onset and disease severity and LOS in AP patients. No studies to date have evaluated the pulse–temperature relationship in AP.

## 2 Materials and methods

### 2.1 Patients

We conducted a retrospective cohort study on consecutive patients admitted with a diagnosis of AP at St. Luke's International Hospital from January 1, 2010, to May 31, 2018. Extensive electronic chart reviews were conducted for each of 275 AP patients. Patients were only included if they were diagnosed with AP on the day of admission and if AP was the main reason for admission. Patients who did not develop fever at any point during the hospital stay were excluded. Those who were either on beta blockers or had implanted cardiac pacemakers were excluded due to their impact on the heart rate. Patients who died during admission, were transferred to another hospital, or were discharged against medical advice were excluded due to the potential impact on LOS. Finally, patients for whom sufficient data was not available were also excluded.

### 2.2 Definitions

Based on the Atlanta classification, AP was diagnosed if at least 2 of the following 3 features were present: characteristic abdominal pain, amylase or lipase elevated to more than 3 times the upper limit of normal, and radiographic evidence of pancreatitis.^[7]^ AP severity was evaluated using the Ranson score, with the modified Ranson score used for biliary AP.^[4,8]^ Contrast-enhanced computed tomography (CT) was performed in all patients without renal disease or known allergies and most were re-evaluated by CT 48 hours after admission. All examinations were evaluated by radiologists for the presence of necrosis or extra-pancreatic complications. CT severity was determined using the modified CT severity index.^[9]^ Delirium was diagnosed based on the Confusion Assessment Method for the Intensive Care Unit.^[10]^

**Figure 1:**
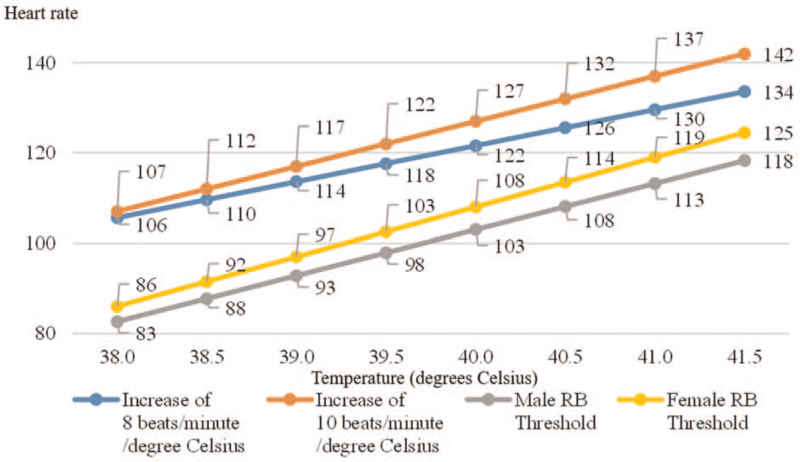
Cutoff heart rates for relative bradycardia. Expected heart rates at various temperatures above 38.0 °C (assuming increases of 8 or 10 beats per minute per °C) are plotted against the calculated cutoffs for relative bradycardia for each gender. RB = relative bradycardia.

Fever was defined as an axillary temperature of at least 38.0 °C at any moment during the hospital stay, measured by an electronic thermometer. Blood and urine cultures were taken from all patients who developed fever. Patients were divided into the RB group and the nonbradycardia (NB) group based on their heart rate at the first instance of fever during the hospitalization. Patients with heart rates less than the expected heart rates calculated using the formula devised by Ostergaard et al^[11]^ at fever onset were considered to have RB. Specifically, male patients with heart rates < 10.2 × body temperature (Celsius) – 305 and female patients with heart rates < 11 × body temperature (Celsius) – 332 were placed in the RB group (Fig. 1). Postfever LOS was defined as the number of days from the onset of the first instance of fever to the date of discharge.

### 2.3 Treatment

All patients received aggressive hydration, with total volumes adjusted for patients with cardiovascular or renal disorders. Most patients without contraindications received antibiotics, proton pump inhibitors, and protease inhibitors in accordance with the clinical pathways of the hospital during the study period. Analgesics with or without antipyretic properties were given as needed. The date of discharge was determined by the attending physician. Conditions for discharge generally included stable vital signs, absence of fever, normal or decreasing trend in C-reactive protein (CRP), lack of active complications requiring close monitoring, pain-free or almost pain-free status without opioid analgesics, and toleration of a soft diet.

### 2.4 Evaluation

Data on demographics, etiology, clinical variables, and outcomes were extracted for analysis. Denominators were reduced for calculations of percentages and ratios when data were missing. The primary outcome was postfever LOS.

### 2.5 Ethical considerations

This study was approved by the ethics committee at St. Luke's International Hospital (18-J019). Patient consent was waived due to the retrospective study design. An overview of the study was publicized on the hospital website, stating that patients could opt-out of the study freely without any impact on their care.

### 2.6 Statistical analysis

Collected data were compared between the RB and NB groups using Pearson chi-square test for categorical variables and the Mann–Whitney *U* test for continuous variables. Pearson correlation coefficient was calculated to investigate correlation between variables. Multiple regression analysis was performed with postfever LOS as the primary outcome, using factors found to be significant in univariate analysis. *P*-values <.05 were considered statistically significant. All statistical analyses were conducted using IBM SPSS Statistics ver. 25.0 (IBM Corp., Armonk, NY).

## 3 Results

### 3.1 Patient characteristics

**Figure 2:**
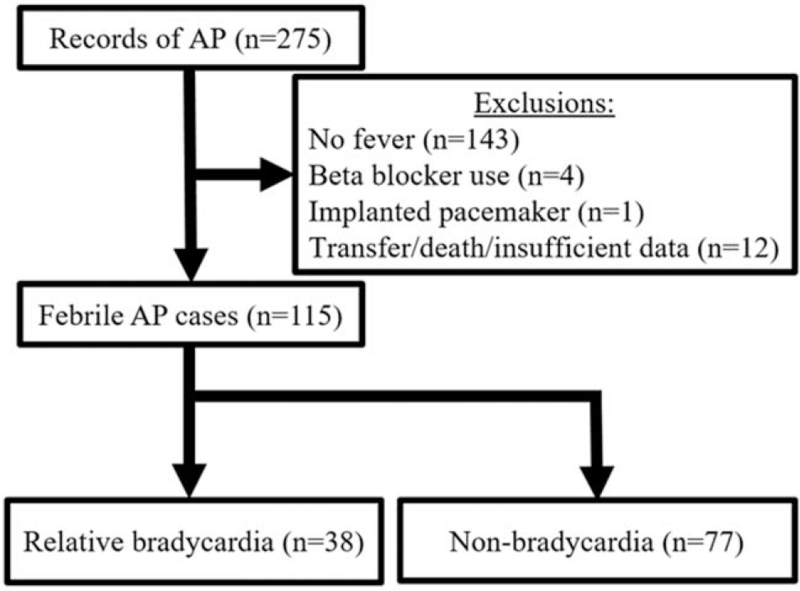
Study flow diagram. AP = acute pancreatitis.

There were 275 admissions for AP during the period. The 143 cases (52.0%) which did not have a temperature of at least 38.0 °C at any point during the hospital stay, 4 receiving beta blockers, one with an implanted cardiac pacemaker, and 12 who died, were transferred to another hospital, were discharged against medical advice, or lacked sufficient data were excluded (Fig. 2). No patients had bradyarrhythmias. Only 3 patients had heart rates below 60 beats per minute on admission; all 3 were confirmed to have sinus rhythm on electrocardiogram.

Fever at or during admission was observed in 115 AP patients. The median age was 53 years (interquartile range: 42–71 years) and 73.9% were male. Etiologies were alcoholic in 47.8%, gallstones in 16.5%, obstructive (pancreatic stones or mass) in 6.1%, drug-induced in 0.9%, trauma-related in 0.9%, and undetermined in 27.8% of cases. Fever was observed at presentation in 47.8% and within 48 hours in 93.9% of cases. The median postfever LOS was 11 days (interquartile range: 7–15 days).

### 3.2 Treatment

All patients received aggressive hydration, with total volumes adjusted for patients with cardiovascular or renal disorders. Antipyretics were given to 86.1% of cases during their hospital stay and 34.8% received antipyretics for pain relief before the onset of fever. Antibiotics were used in 111 (96.5%) cases; 55 (47.8%) were febrile at admission, 53 (46.1%) received antibiotics at admission despite the lack of fever, and 3 received antibiotics for the first time at fever onset postadmission. Antibiotics were changed at fever onset in 66 (57.3%) cases to achieve broader coverage and in consideration of possible drug fever. Postfever LOS was not affected by initiation of, or a change in, antibiotics at fever onset (*P* = .311).

### 3.3 Factors associated with relative bradycardia at fever onset

Of the 115 included patients, 38 patients (33.0%) had RB and 77 (67.0%) were NB (Table 1). Patients in the RB group were older than those in the NB group (median of 62 vs 51 years, *P* = .028). Median postfever LOS was significantly shorter in RB patients (8 vs 12 days, *P* = .003). The RB group had lower median Ranson scores than the NB group (1 vs 2, *P* < 001). Severe AP was observed in 21 cases, of which only 2 were in the RB group (*P* = .011). All 6 cases with positive blood cultures were in the NB group (*P* = .077). Extra-pancreatic infection was suspected in 9 cases (5 with cholangitis, 1 with cholecystitis, 1 with aspiration pneumonia, 1 with catheter-related infection, and 1 with bowel perforation) but cultures were only positive in 3 of these cases. Eight others had possible extra-pancreatic causes of fever, of which 3 had positive blood cultures with no clearly identified source and 5 were noninfectious (gout, pseudogout, pulmonary embolism, and deep vein thrombosis). There was no significant difference between the 2 groups with respect to suspected extra-pancreatic infection or causes of fever.

**Table 1:** Patient characteristics.

	All Febrile AP (n = 115)	Relative Bradycardia (n = 38)	Nonbradycardia (n = 77)	*P*-value
Age (yr), median [IQR]	53 [42–71]	62 [47–74]	51 [40–70]	.028^∗^
Male, n (%)	85 (73.9)	30 (78.9)	55 (71.4)	.388
Body mass index, median [IQR]	23.1 [21.1–27.4]	23.0 [21.1–28.5]	23.2 [21.2–27.4]	.747
Etiology (alcoholic/biliary/other), n (%)	55/19/41 (47.8%/16.5%/35.7%)	16/6/16 (42.1%/15.8%/42.1%)	39/15/26 (50.6%/16.9%/33.8%)	.772
Ranson score, median [IQR]	1 [1–2]	1 [0–1]	2 [1–2]	<.001
Severe AP (Ranson score), n (%)	21 (18.3)	2 (5.3)	19 (24.7)	.011^∗^
Recurrent AP, n (%)	19 (16.5)	10 (26.3)	9 (11.7)	.085^∗^
Fever at admission, n (%)	55 (47.8)	18 (47.4)	37 (48.1)	.945
Body temperature at admission, median [IQR]	36.9 [36.3–37.3]	36.9 [36.3–37.2]	36.8 [36.2–37.3]	.681
Body temperature at fever onset, median [IQR]	38.2 [38.1–38.4]	38.2 [38.1–38.5]	38.2 [38.1–38.4]	.530
Heart rate at fever onset, median [IQR]	96 [82–111]	77 [71–82]	104 [96–119]	<.001^∗^
Pain at fever onset (NRS), median [IQR]	3 [0–4]	3 [0–4.5]	3 [0–4]	.945
ERCP performed, n (%)	29 (25.2)	13 (34.2)	16 (20.8)	.119
Delirium, n (%)	33 (28.7)	4 (10.5)	29 (37.7)	.002^∗^
Blood culture positivity, n (%)	6 (5.2)	0 (0)	6 (7.8)	.077
Antipyretics used before fever onset, n (%)	40 (34.8)	14 (36.8)	26 (33.8)	.745
Postfever LOS (d), median [IQR]	10 [7–14]	8 [6–11]	12 [8–15]	.003^∗^
Total LOS (d), median [IQR]	11 [7–15]	9 [7–11]	12 [8–16]	.003^∗^
Fever duration (days), median [IQR]	2 [1–3]	1.5 [1–2]	2 [1–4]	.074
Fluid sequestration > 5 L at 48 h, n (%)	36 (31.3)	6 (15.8)	30 (39.0)	.012^∗^
Fluid output > input at 48 h, n (%)	6 (5.2)	5 (13.2)	1 (1.3)	.007^∗^
Necrosis on CT, n (%)	14 (12.2)	1 (2.6)	13 (16.9)	.028^∗^
Modified CTSI: moderate or severe AP, n (%)	39 (33.0)	8 (21.1)	31 (40.3)	.041^∗^
Modified CTSI: severe AP, n (%)	11 (9.6)	1 (2.6)	10 (13.0)	.076
Extra-pancreatic complications on CT, n (%)	21 (18.3)	7 (18.4)	14 (18.2)	.975

AP = acute pancreatitis, CI = confidence interval, CT = computed tomography, CTSI = CT severity index, ERCP = endoscopic retrograde cholangiopancreatography, IQR = interquartile range, LOS = length of stay, NRS = numerical rating scale.

∗ Statistically significant difference between relative bradycardia and nonbradycardia groups (*P* < .05).

Delirium was observed in 28.7% of cases and was less commonly observed in the RB group (10.5% vs 37.7%, *P* = .002). While there was a tendency for recurrent AP patients (admitted 2–7 times) tended to have RB (*P* = .085), patients with RB in 1 AP episode did not necessarily have RB in the next recurrence.

Necrosis on CT was observed in 13 (16.9%) NB cases but in only 1 (2.6%) RB case (*P* = .028). Moderate or severe AP based on the modified CT severity index was observed more commonly in the NB group. Twenty-two extra-pancreatic complications were observed on CT in 19 patients, including 14 pancreatic pseudocysts and 5 portal or splenic vein thromboses. There was no significant difference in CT complications between the 2 groups.

**Figure 3:**
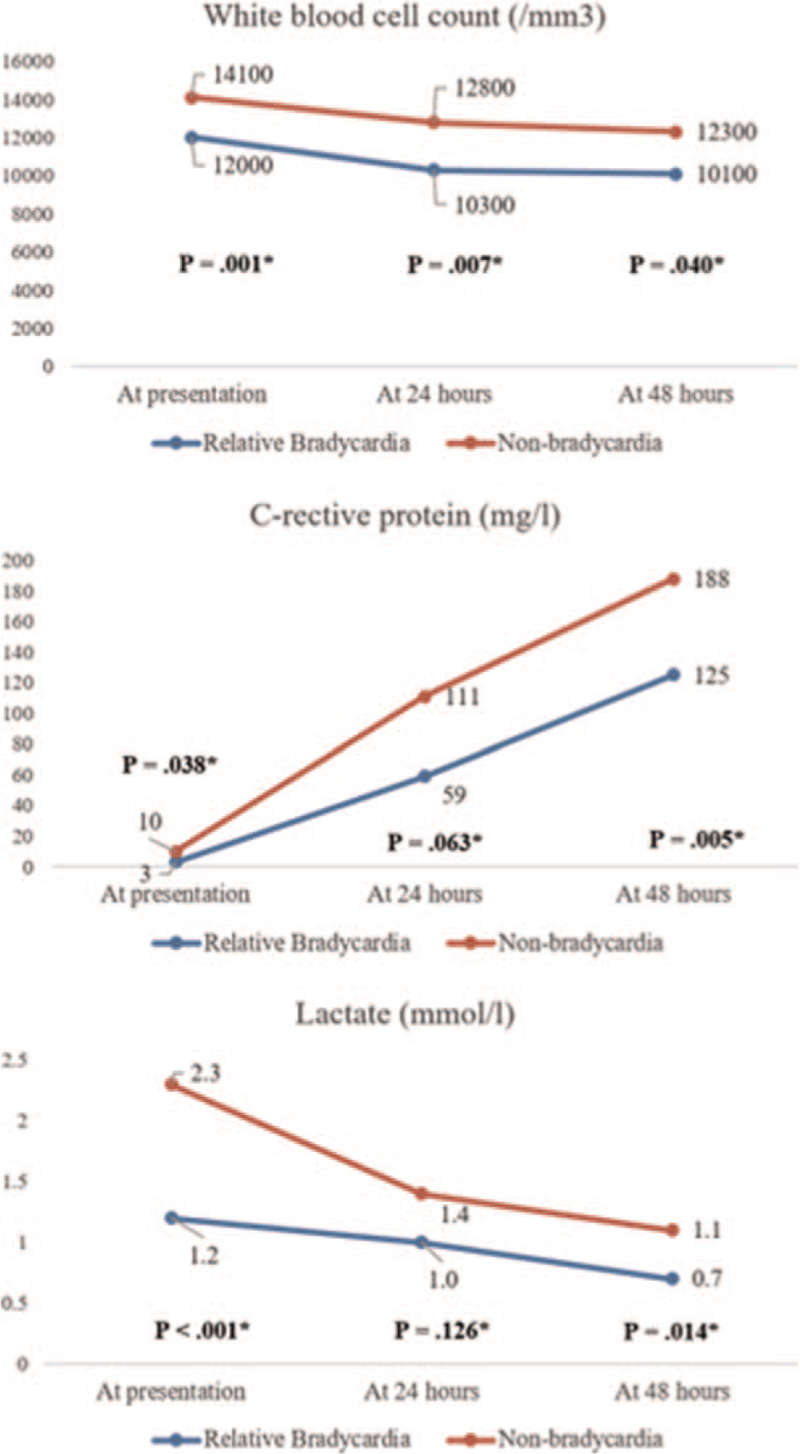
Line graphs illustrating differences in white blood cell count, C-reactive protein, and lactate levels between relative bradycardia and nonbradycardia patients during the first 3 days of admission.

White blood cell count, CRP, and lactate levels were significantly lower in the RB group (Fig. 3). Lower white blood cell count (*P* = .001) and lactate (*P* < .001) were most significantly associated with RB at admission. CRP after 48 hours, which has been reported as the most accurate predictor of severity,^[12]^ had a lower *P*-value (*P* = .005) than CRP measured at earlier times.

RB had a favorable odds ratio of 0.17 (95% confidence interval: 0.04–0.77) for AP severity based on the Ranson score. There was a significant inverse correlation between RB and the Ranson score (−.305, *P* = .001).

Linear regression analyses revealed that RB as well as age, need for endoscopic retrograde cholangiopancreatography (ERCP), recurrent AP, necrosis and extra-pancreatic complications on CT, and the Ranson score were significant predictors of post-fever LOS (Table 2). AP severity based on the Ranson score and the fluid sequestration component of the Ranson score were also significant. Multiple regression analysis revealed that RB was an independent predictor of postfever LOS after adjusting for all other factors excluding the Ranson score (age, need for ERCP, recurrent AP, necrosis and extra-pancreatic complications on CT) (*P* = .010). RB ceased to be significant when the Ranson score was added to the analysis (*P* = .141).

**Table 2:** Regression analysis of factors affecting postfever length of stay.

	Linear regression			Multiple regression (excluding Ranson score)			Multiple regression (including Ranson score)		
	Beta coefficient	95% CI	*P*-value	Beta coefficient	95% CI	*P*-value	Beta coefficient	95% CI	*P*-value
Age	0.15	.03∼.24	.017^∗^	0.16	.05∼.28	.006^∗^	0.10	−.02∼.22	.091
Relative bradycardia at fever onset	−4.53	-8.93 ∼−.15	.043^∗^	−5.21	−9.14∼−1.29	.010^∗^	−3.05	−7.12∼1.03	.141
ERCP performed	4.94	.18 ∼ 9.70	.042^∗^	2.91	−1.46∼7.29	.189	2.45	−1.79∼6.69	.213
Recurrent AP	−5.59	−9.91∼−1.27	.032^∗^	−2.44	−6.27∼1.39	.209	−2.34	−6.05∼1.36	.213
Necrosis on CT	7.71	1.43∼13.98	.016^∗^	3.74	−1.89∼9.37	.191	2.09	−3.47∼7.65	.458
Extra-pancreatic complications on CT	13.6	8.19∼17.93	<.001^∗^	12.13	7.47∼16.8	<.001^∗^	10.64	6.01∼15.26	<.001^∗^
Ranson score	4.89	3.17∼6.61	<.001^∗^				2.72	.86 ∼ 4.58	.005^∗^
*Severe AP (Ranson score)*	*11.9*	*6.92∼16.87*	*<.001* ^∗^						
*Fluid sequestration >5 L in 48 h*	*8.55*	*4.31∼12.80*	*<.001* ^∗^						
				(Adjusted R^2^ = .313, *P* < .001)			(Adjusted R^2^ = .357, *P* < .001)		

AP = acute pancreatitis, CI = confidence interval, CT = computed tomography, ERCP = endoscopic retrograde cholangiopancreatography.

∗ Statistically significant predictor of postfever length of stay (*P* < .05).

## 4 Discussion

Our results suggest that the heart rate at fever onset may predict lower severity and shorter postfever LOS in AP patients. RB is a simple alternative to the various scoring systems available to predict AP severity. The presence of RB can often be determined before the Ranson score can be calculated, as over 90% of fevers in AP patients occurred within the first 48 hours of admission.

### 4.1 Fever and bradycardia (relative bradycardia)

An increase in the heart rate of 8 to 10 beats per minute is associated with every degree Celsius of increase in body temperature, in a pulse–temperature relationship also known as Liebermeister rule.^[13]^ RB, or pulse–temperature dissociation, refers to an increase in body temperature without the expected corresponding increase in the heart rate. Various infectious etiologies such as bacteria (including Chlamydia, Listeria, Rickettsia, Legionnaire disease, typhoid fever, Q fever, tularemia, psittacosis, Rocky Mountain spotted fever, Lyme disease, and mild to moderate conoravirus disease 2019), viruses, and parasites have been reported, as well as noninfectious etiologies including drug fever, lymphoma, adrenal insufficiency, cyclic neutropenia, and factitious fever.^[14,15]^ The mechanisms underlying RB are not well understood but have been suggested to include pathogenic impact on the sinoatrial node or myocardium, inflammatory cytokine release, increased parasympathetic stimulation, and reduced heart rate variability.^[14,16]^ The pulse–temperature relationship is most sensitive for temperatures above 38.9 °C.^[13,17]^

Given these proposed mechanisms, RB can theoretically occur in the setting of many other etiologies. RB may be underreported due to the lack of physician awareness and the lack of a consistent definition. The formula used in our study comes from a Danish study on 6000 febrile patients which defined RB as heart rates below the lower limit of the 95% confidence interval.^[11]^ In our analysis, we defined RB as heart rates below the expected heart rates according to the formula, which are already much lower than expected heart rates under traditional definitions.

A study on septic shock requiring vasopressor therapy found that patients with RB (44% of the sample) were more likely have lower mortality and slightly lower severity, despite being older.^[18]^ While the study defined RB as a heart rate of less than 80 beats per minute in the presence of septic shock with no reference to temperature, the results are similar to ours in that older patients were more likely to have RB and RB patients had better outcomes.

### 4.2 Fever and Pancreatitis

Fever has been reported to occur in 31% to 85% of AP cases and was observed in 48% of our initial sample.^[19,20]^ Etiologies of fever in AP include pancreatitis itself, pancreatic or peripancreatic infection (generally observed in the setting of necrotizing pancreatitis), extra-pancreatic infection (including concomitant cholangitis in gallstone pancreatitis, catheter-related infections, and aspiration pneumonia), fever caused by the causative infectious agent in bacterial, viral, fungal, or parasitic pancreatitis, and drug fever.^[19,21,22]^ Bohidar et al^[19]^ analyzed 45 febrile AP patients and concluded that infected pancreatic necrosis was the cause of fever in only 18% of cases. Another report found that inflammation per se was the cause of fever in 57.7% of febrile AP patients and that prolonged fever of over 2 days was associated with higher severity, pancreatic necrosis, sepsis, and higher CT grades and CRP.^[20]^

Pancreatic infection, the leading cause of death in late-stage severe AP, was found in 12% of 1741 cases in a systematic review.^[22,23]^ Bacterial translocation has been implicated as the main cause of pancreatic infection, which may be difficult to prove due to the low sensitivity of traditional blood cultures.^[24,25]^ The same review found extra-pancreatic infections in 32% of AP cases, most commonly respiratory infection and bacteremia, which were not associated with predicted survival or mortality.^[22]^ Pancreatic infections may be associated with preceding or simultaneous extra-pancreatic infections.^[26]^ Regardless of the infectious source, culture positivity has been linked to greater mortality and combined pancreatic and extra-pancreatic sources of sepsis are associated with longer hospital stays.^[27]^ On the other hand, randomized control trials have failed to demonstrate the efficacy of prophylactic antibiotics in AP.^[27]^

### 4.3 Pancreatitis and Bradycardia

Approximately 50% of AP patients have abnormal findings on electrocardiogram which generally resolve upon recovery, with atrioventricular block and bradyarrhythmias in rare cases.^[28–30]^ The relationship between fever and heart rate has not been studied in the context of pancreatitis. None of our patients were newly diagnosed with atrioventricular block or bradyarrhythmia. As RB patients were significantly older than NB patients in our study, the decrease in heart rate variability in the elderly may have played a role.^[30]^ While mean heart rates are not significantly related to age or gender, minimum heart rates are higher and maximum heart rate are lower in the elderly, particularly elderly men.^[30,31]^ On the other hand, RB remained significant when adjusting for age in our multiple regression analysis.

RB cases may lack the various causes of tachycardia that may occur in febrile AP. Over 90% of severe or necrotic AP cases and all cases with positive blood cultures were in the NB group. As intravascular fluid depletion is another potential cause of tachycardia, fluid sequestration of exceeding 5 L at 48 hours was more common in the NB group (15.8% vs 39.0%, *P* = .012) and urine output exceeded fluid input at 48 hours was more common in the RB group (13.2% vs 1.3%, *P* = .007). Absent bacterial infection, fluid depletion, severe AP which may cause excessive cytokine release, and delirium, fever due to pancreatitis per se may follow a relatively benign course.

### 4.4 Length of stay

LOS depends on various factors, including the country of admission. Median LOS in the United States is 3.8 days, but about 8 days in Spain and Italy.^[32–34]^ Our median LOS in all AP patients before exclusions was 8 days, similar to European reports.

In our study, linear regression analyses predictably indicated that older age, need for ERCP, necrosis or extra-pancreatic complications on CT, and higher Ranson scores were associated with longer postfever LOS. Both RB and recurrent AP were significant predictors of shorter postfever LOS. As patients with recurrent AP were admitted 2 to 7 times, they may recognize early signs of recurrence, allowing them to present early and receive timely treatment.

Along with age and extra-pancreatic complications on CT, RB remained a significant predictor of postfever LOS in multiple regression analysis when adjusting for all significant factors from linear regression analysis, with the exception of the Ranson score. The significant negative correlation between RB and the Ranson score was probably the main reason that RB ceased to remain significant when the Ranson score was added. On the flipside, this negative correlation supports RB as a favorable prognostic indicator in febrile AP patients. Furthermore, fever was observed within 48 hours in 93.9% of cases. The presence of RB can therefore be evaluated before the Ranson score can be calculated in most cases, serving as an early clue to a favorable prognosis. As there are naturally limits to predicting LOS based on a single dichotomous variable, RB should be considered together with other clinical factors.

There are several limitations to this study. The study was a retrospective study at a single institution with a limited sample size, limiting the generalizability of study results. Patients were determined to have fever based on any single measurement above 38.0 °C, which may have been transient and unrelated to pancreatitis. Grouping of patients into RB and NB groups were influenced by timing of measurement and chance, as heart rates and body temperatures change constantly. The formula used to calculate RB may not be applicable to our sample of Japanese patients. Nonmedical factors such as patient requests for early or delayed discharge may have affected LOS. Only patients on beta blockers were excluded; patients on calcium channel blockers, digoxin, or other medications which may affect the heart rate were not excluded. However, these other medications have a less significant impact on the pulse–temperature relationship compared to beta blockers.^[17]^ Drug fever, a reported cause of RB, is always difficult to rule out. Many patients were also given acetaminophen for pain relief and/or prophylactic nonsteroidal anti-inflammatory drugs after ERCP, which may have reduced body temperatures as well as heart rates.

## 5 Conclusion

RB at fever onset were associated with older age but with lower Ranson scores, less delirium, and shorter postfever LOS. RB may be a useful early predictor of a favorable clinical course, particularly when fever arises before the Ranson score can be calculated. Multicenter studies with larger sample sizes are desirable to further investigate the significance of RB in febrile AP patients.

## Author contributions

**Conceptualization:** Takeshi Okamoto.

**Formal analysis:** Takeshi Okamoto, Ryosuke Tsugitomi.

**Investigation:** Makoto Arashiyama.

**Methodology:** Takeshi Okamoto, Ryosuke Tsugitomi.

**Supervision:** Takeshi Okamoto, Kenji Nakamura, Ryosuke Tsugitomi, Katsuyuki Fukuda.

**Validation:** Takeshi Okamoto.

**Writing – original draft:** Takeshi Okamoto.

**Writing – review & editing:** Takeshi Okamoto, Makoto Arashiyama, Kenji Nakamura, Ryosuke Tsugitomi, Katsuyuki Fukuda.
